# The Great Spiral Masquerader: A Case of Concurrent Secondary Syphilis and Autoimmune Hepatitis

**DOI:** 10.14309/crj.0000000000000451

**Published:** 2020-09-29

**Authors:** Michelle Baliss, Kevin Kline, Kashif Khan, Heather L. Stevenson

**Affiliations:** 1 Department of Internal Medicine, The University of Texas Medical Branch, Galveston, TX; 2 Division of Gastroenterology & Hepatology, The University of Texas Medical Branch, Galveston, TX; 3 Department of Pathology, The University of Texas Medical Branch, Galveston, TX

## Abstract

We describe a unique case of rash and acute hepatitis confounded by the presence of syphilis that created suspicion for syphilitic hepatitis, a rare and often misdiagnosed condition. Investigation concerning the etiology alternatively lead to the diagnosis of 2 concomitant conditions: active autoimmune hepatitis and secondary syphilis. To our knowledge, this is the first description in the literature of the simultaneous occurrence of secondary syphilis and autoimmune hepatitis. This case serves to increase the recognition of the clinical characteristics and diagnostic challenges of syphilitic hepatitis and to discuss the potential role of pathogens in the induction of autoimmunity.

## INTRODUCTION

Syphilis manifests with varying nonspecific symptoms that make it diagnostically challenging and renders the recognition of concurrent diagnoses quite difficult. Infrequently, it can present as syphilitic hepatitis (SH) by spirochete dissemination to the liver, a diagnosis that requires exclusion of other causes of liver injury.^1,2^ Autoimmune hepatitis (AIH) pathogenesis is speculated to involve the interplay of genetic predisposition and failed immune tolerance because of triggers such as infections; however, there is a paucity of evidence that pathogens can initiate or propagate autoimmune responses.^3,4^ Despite this, the ability of *Treponema pallidum* to activate autoimmune responses has been demonstrated in natural and experimental syphilis, raising the question of its implication in the etiology of autoimmune diseases.^5^

## CASE REPORT

**Figure 1.**
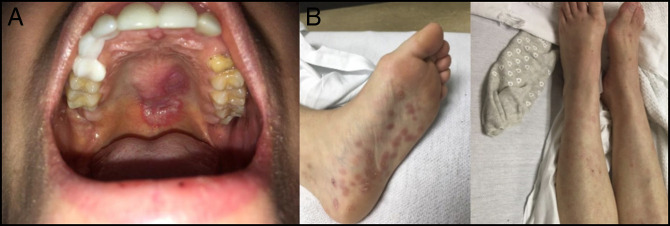
(A) Tender oral ulcer and (B) rash involving bilateral soles and torso.

**Figure 2.**
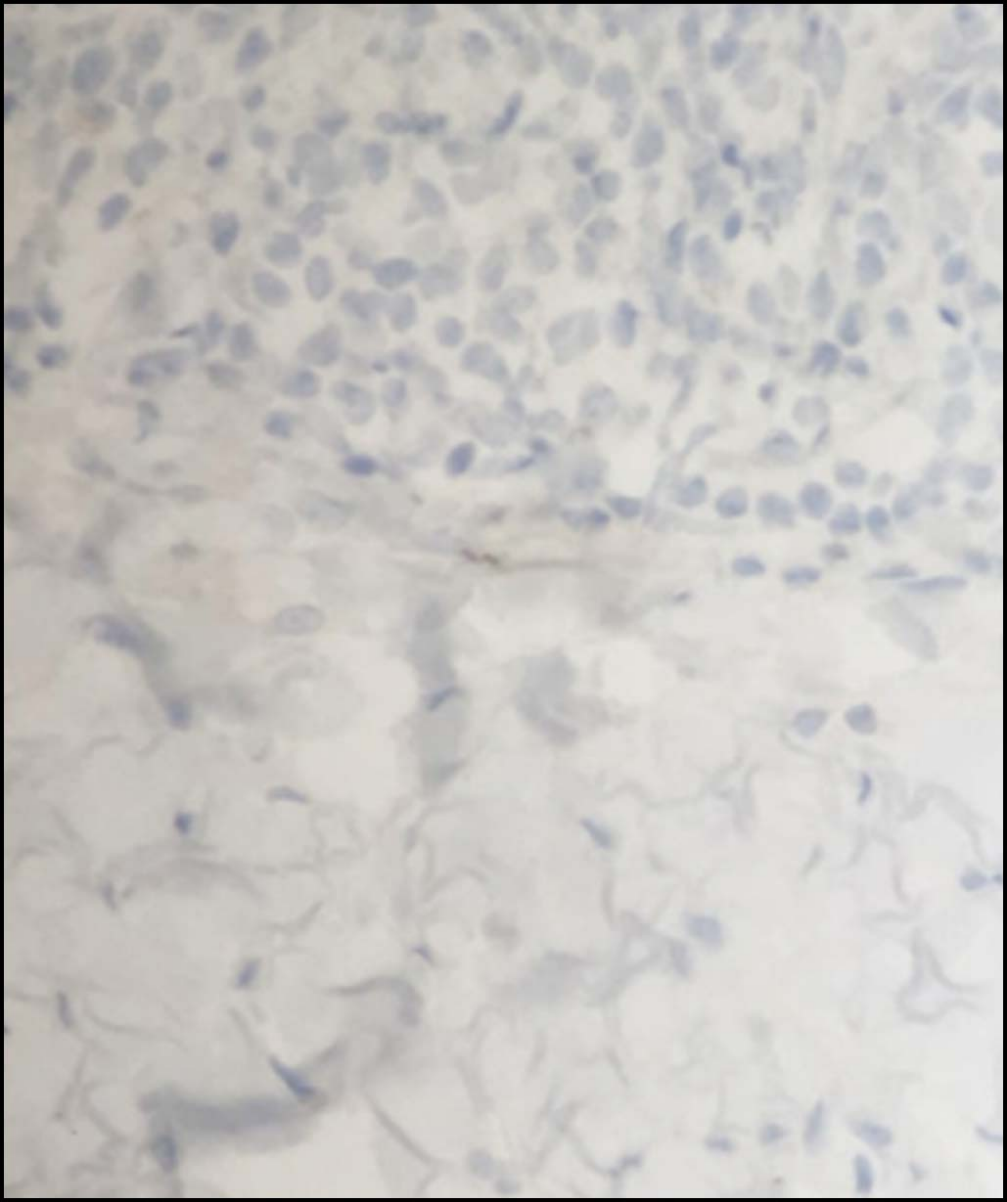
Rash biopsy showing the presence of spirochetes.

A 22-year-old incarcerated woman presented to the clinic with 3 weeks of right-sided abdominal pain, rash, vomiting, and jaundice. Vital signs were normal. Physical examination revealed a tender erosion on her soft palate, jaundice, right-sided abdominal tenderness, and a maculopapular rash involving her soles and torso (Figure 1). Laboratory work was significant for alanine aminotransferase (ALT) of 1,900 U/L, aspartate aminotransferase (AST) of 1,674 U/L, bilirubin of 7.1 mg/dL (predominantly conjugated), and alkaline phosphatase (ALP) of 168 U/L. Magnetic resonance cholangiopancreatography showed hepatomegaly with normal contour, periportal edema, and two 1.8 cm areas of early enhancement that may reflect hepatocellular damage. Serologies for viral hepatitis, autoimmune conditions including lupus, human immunodeficiency virus, and Wilson disease, and connective tissue diseases were unrevealing. There was a high degree of nonspecific fluorescence for liver-kidney-microsome antibody, and mitochondrial antibody testing was equivocal at 23.3 U. Antinuclear antibody was detected at 1:160 titer in a speckled pattern. Immunoglobulin G (IgG) levels were 1,610 mg/dL. Smooth muscle IgG was within the normal limits. Rapid plasma reagin was positive with a titer of 1:512 with reactive treponemal particle agglutination and reactive syphilis IgG/immunoglobulin M. Pedal rash biopsy showed findings consistent with secondary syphilis (SS), further supported by *T. pallidum* spirochetes on immunohistochemical stains (Figure 2).

**Figure 3.**
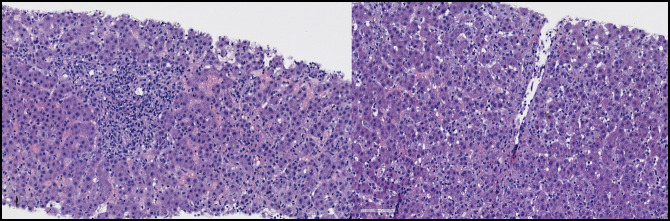
Liver biopsy showing the features of autoimmune hepatitis with lymphoplasmacytic portal inflammation, plasma-rich cell interface, and perivenulitis.

Treatment with intramuscular penicillin G interestingly resulted in improvement in ALT to 1500 U/L and AST to 1,000 U/L, raising suspicion for SH; however, this improvement came to a plateau despite therapy completion, prompting investigation for an alternative etiology. Liver biopsy showed active interface hepatitis with lymphoplasmacytic portal inflammation, plasma cell-rich interface activity, suggestive of AIH with negative spirochete staining (Figure 3). Prednisone initiation resulted in a dramatic improvement in symptoms and biochemical abnormalities, strengthening the case for AIH. She was discharged on a prolonged steroid taper. On a 5-week follow-up, liver chemistries had improved (ALT 139 U/L, AST 86 U/L, ALP 45 U/L, and bilirubin 0.9 mg/dL). Azathioprine was started, and prednisone taper was continued. Five weeks later, rapid plasma reagin titer was negative, and liver chemistries were normal.

## DISCUSSION

Syphilis often poses diagnostic challenges by mimicking features of other diseases. It progresses from the primary form of a self-limiting chancre to its secondary form characterized by low-grade fevers, lymphadenopathy, weight loss, and a distinctive rash.^6^ Lack of symptom specificity often results in misdiagnosis. Left untreated, syphilis can enter an asymptomatic latent stage and remain unrecognized for decades, allowing for disease transmission and progression to tertiary syphilis with severely detrimental consequences. It is therefore crucial to recognize its uncommon manifestations.

SH is extremely rare with only 3% presenting with clinical hepatitis.^2^ Biochemically, it is characterized by a disproportionate elevation in ALP and GGT. A systemic review of 99 patients showed a mean AST of 253 U/L, ALT of 314 U/L, ALP 684 U/L, and GGT at 562 U/L. AST and ALT levels in the 1,000s have not been reported previously in SH. Histologic findings of SH include pericholangiolar inflammation, periportal necrosis, and occasionally spirochetes.^7^ Among 28 cases, spirochetes were detected in only 19 cases, believed to be because of Kupffer cell phagocytosis.^2^ A diagnosis of SH can therefore be established by the proposed criteria of elevated liver enzymes, seropositive testing for syphilis, a presentation consistent with SS, absence of other diagnoses, and improvement with appropriate antibiotics.^8^

This case highlights the importance of assessing for alternative diagnoses that may be overlooked with syphilis. In this case, the initial suspicion of AIH was confounded by the presence of SS. The laboratory disturbances could have been explained by an intrahepatic syphilis infection, cytokine-mediated processes driven by extrahepatic syphilis, or an alternative condition which needed to be excluded before the presumption of SH. It became difficult to define the etiology in this patient based solely on laboratory findings. The hypothesis of SH was ultimately refuted by the biopsy confirmation of AIH.

Although the laboratory workup was not definitive for AIH, the clinical picture and biopsy results suggested otherwise. The elevation of transaminases did not coincide with the typical SH pattern. Transaminase improvement with antibiotics was minimal despite treatment completion. It was not until steroid initiation that a substantial improvement occurred. Thus, the presence of 2 concurrent diagnoses of AIH and SS was concluded. Multiple scoring systems for AIH diagnosis exist and are based on typical histological, clinical, and laboratory findings, elevated IgG, characteristic autoantibodies, and absence of viral markers.^9^ The histological hallmark of AIH is interface hepatitis and plasma cell infiltration, as seen in this case.^10^

The potential association of infection with AIH has been described extensively, but causation has been difficult to establish.^4^ Although the presence of AIH and SS could be incidental, it is feasible to consider a potential association, perhaps through pathogen-mediated breakdown of immunological self-tolerance in the liver and activation of autoimmunity in genetically susceptible individuals. Molecular mimicry is a process commonly observed in *T. pallidum*, whereby a pathogen drives proinflammatory responses against host tissue through pre-existing or disguised structural homology with host antigens.^5^ Postinfectious autoimmunity can also occur through the alteration of the immunoregulatory system by inappropriate upregulation of self-reactive T-cells. It is also proposed that tissue destruction from the infection alone can induce autoantibody production if host proteins are sufficiently altered by the offending organism.^5^ Studies regarding immunologic phenomena in *T. pallidum* predominantly explore the humoral immune response that results in the well-known production of various autoantibodies. However, emerging evidence from animal studies suggests the involvement of the cellular arm of the immune response through transient sensitization of lymphocytes by the pathogen to homologous organ antigens.^5^ It is also possible that a small number of spirochetes can remain in the host and reactivate previously sensitized T-cells, leading to an amplified tissue destructive process.^11^ Unfortunately, the immunopathologic processes in *T. pallidum* remain poorly understood with an overall lack of solid evidence implicating pathogenic triggers for AIH. This case is unique in that it represents the first report of simultaneous AIH and SS and emphasizes the importance of reporting such cases to improve understanding of postinfectious autoimmunity. It also highlights the importance of entertaining SH as a potential cause of hepatitis in the proper setting to minimize avoidable transmission.

## DISCLOSURES

Author contributions: M. Baliss and K. Kline wrote the manuscript. K. Khan and H. Stevenson edited the manuscript. M. Baliss is the article guarantor.

Financial disclosure: None to report.

Informed consent was obtained for this case report.
